# Highlight: A Matter of Taste—Whales Have Abandoned Their Ability to Taste Food

**DOI:** 10.1093/gbe/evu107

**Published:** 2014-06-02

**Authors:** Danielle Venton

Living in a world of prepared salads, soups, and sandwiches, our survival may not seem to hinge upon careful tasting what we eat. But for most of our species' history, it was a life or death matter, and—for most other animals—it still is. Their sense of taste keeps them alive.

"The most significant decision you make in your life everyday is, when you put something into your mouth, whether to swallow it or reject it,” says Gary Beauchamp, director and president of the Monell Chemical Senses Center in Nashville, TN.

It is a puzzle then why whales have, by and large, lost their sense of taste, as reported in a new study published in *Genome Biology Evolution* (Feng et al. 2014).

"I was really surprised by what we found. I think most people were,” says coauthor Huabin Zhao, a zoologist at Wuhan University in China. “We didn't expect such a big group of mammals could afford to lose almost all taste sense.”

Zhao became interested in examining whale taste in 2012 when an article reported massive taste loss in several carnivorous mammals including the bottle-nosed dolphin. “I thought [for marine mammals] the dolphin would be an isolated case,” he says.

Instead, in all 12 examined whales—five species of baleen whales and seven species of toothed—they found the sweet, umami, and bitter tastes were gone. The genes needed to code for taste receptors were “pseudogenes”—mutated past the point of functionality. They checked, based on the integrity of sour taste genes, the ability to taste sour things in three whales and found it missing in all of them. (Whether whales can taste salt is not clear at the moment. Though genetic analysis shows salty taste receptor genes are evolutionarily conserved in toothed and baleen whales, these same receptor genes control sodium balance in kidneys and may not be functional in taste.)

The loss of most, if not all, of their tasting ability challenges long-held assumptions. “That these animals can still be alive,” Zhao says, “is the most surprising part.”

The five taste sensations found in mammals: Sweet, umami, bitter, sour, and salty, each play a fundamental role meeting nutritional needs. Sweet tastes signal carbohydrates, a crucial source of calories for many animals. Umami is generally found alongside food rich in amino acids, which are needed to build protein. Salt—particularly the sodium in sodium chloride—is essential to many physiological processes, such as the transmission of nerve impulses. Strong sour flavors may signal spoiled food. Bitter flavors almost universally indicate toxicity. Though insects diverged from mammals almost a billion years ago, both groups share the same fundamental principles for sensing taste.

Among the vertebrates, taste receptors likely originated in fish, which makes the loss of taste in marine mammals all the more surprising. “Fish probably have a similar diet as some of the marine mammals,” says David Yarmolinsky, of Columbia University, who was not involved in the research. “It's surprising that one whole set of marine predators would have taste receptors, whereas another, which had them to begin with, decided that they were dispensable.”

When did this near-wholesale abandonment of taste occur? Possibly, Zhao and coauthors speculate, when whale ancestors returned to water and made the switch from eating plants—rich sources of sweet, sour, and bitter tastes—to eating meat.

Although no other group of animals seem to have given up their sense of taste so completely, in many corners of the animal kingdom tasting ability does clearly reflect dietary concerns. Cats cannot taste sweet flavors, for example. Their calories come from meat not carbohydrates. The panda bear, which is now vegetarian, has lost its taste for umami.

An interesting exception among marine mammals is the manatee. Unlike whales and dolphins, they have retained the ability to detect sweet tastes, probably because they eat plants. “So it’s not a necessary result of mammals going back to the sea to lose taste receptors,” Beauchamp says. “It depends on what their diet is as they go back.”

However, if you are in the habit of swallowing things whole as many whales do, without chewing or savoring, says Beauchamp, then you might not need to be guided by taste. Nonetheless “they seem to have developed different ways of identifying food, either visually or tactilely or by something we don't know.”

This question is a fascinating one, says Zhao. “We don't know how these marine mammals can survive without the taste function,” he says. “I'm planning to examine the other senses, such as echolocation and olfaction, to see if these can compensate for the loss of taste.”
